# Transcriptomic analysis of bisphenol AF on early growth and development of zebrafish (*Danio rerio*) larvae

**DOI:** 10.1016/j.ese.2020.100054

**Published:** 2020-08-05

**Authors:** Rongzhen Li, Shuai Liu, Wenhui Qiu, Feng Yang, Yi Zheng, Ying Xiong, Guanrong Li, Chunmiao Zheng

**Affiliations:** a State Environmental Protection Key Laboratory of Integrated Surface Water-Groundwater Pollution Control, School of Environmental Science and Engineering, Southern University of Science and Technology, Shenzhen, 518055, China; b Guangdong Provincial Key Laboratory of Soil and Groundwater Pollution Control, School of Environmental Science and Engineering, Southern University of Science and Technology, Shenzhen, 518055, China; c School of Environmental and Chemical Engineering, Shanghai University, Shanghai, 200444, China; d Shenzhen Municipal Engineering Lab of Environmental IoT Technologies, Southern University of Science and Technology, Shenzhen, 518055, Guangdong Province, China

**Keywords:** Bisphenol AF, Zebrafish, Early development, Oxidative stress, MAPK signaling Pathway

## Abstract

Bisphenol AF (BPAF), an alternative to bisphenol A, is widely detected in aquatic environments. Owing to health concerns, the toxic effects of BPAF on organisms are drawing attention. The present study aims to evaluate the toxicity of BPAF, combining the results of omics techniques and experiment. Employing transcriptome sequencing (RNA-seq), we obtained 391, 648, 512, and 545 differentially expressed genes (DEGs) in 0.1, 1, 10, and 100 μg/L BPAF-exposed zebrafish larvae, respectively. Gene ontology (GO) analysis and Kyoto Encyclopedia of Genes and Genomes (KEGG) enrichment revealed the early development, stimulus-response, and MAPK signaling pathway were significantly affected by BPAF. In addition, five hub genes (*fgf3*, *fgf4*, *map2k1*, *myca*, and *casp3b*) were highlighted as the key genes in MAPK signaling pathway using the protein-protein interaction network. Therefore, the RNA-seq results showed that early development and stimulus-response were the main processes affected by BPAF, which was consistent with our morphological and pathological results. The hatching rate of zebrafish embryos in 1 and 10 μg/L BPAF groups was significantly inhibited, and the oxidative stress indexes, including the level of total antioxidant capacity (T-AOC), superoxide dismutase (SOD), and lipid peroxidation (LPO), were significantly increased by the 100 μg/L BPAF treatment. Moreover, the activity of alkaline phosphatase (AKP) was significantly decreased in all BPAF exposure groups. In conclusion, exposure to BPAF at environmental relevant concentrations affected the early development and immune system of zebrafish larvae by modulating MAPK signaling pathway, and our results provide solid evidence for the future studies on the toxicity of bisphenols.

## Highlights

• Bisphenol AF changed the expression of several genes in exposed zebrafish larvae.
• The early development and immune process of zebrafish larvae were disrupted by BPAF treatments.
• The MAPK signaling pathway in zebrafish larvae was severely disrupted by BPAF.
• The hub genes in the MAPK signaling process were *fgf3*, *fgf4*, *map2k1*, *myca*, and *casp3b.*

## 1 Introduction

Bisphenol AF (BPAF) is a substitute of bisphenol A (BPA) and has widespread applications as a monomer and crosslinking reagent in plastic production [1], such as polycarbonate copolymers, food-contact polymers, and electronic materials. Due to its wide use in industry, BPAF has become an emerging environmental pollutant [2]. It was detected in several different environmental matrices near chemical-producing areas in Jiaxing city, including water, sediment, and soil (dust) [3], and the highest concentrations of BPAF in river waters was 15.3 μg/L with a median concentration at 3.08 μg/L. In addition, BPAF was also found in the Liaohe River and Taihu Lake at concentrations of 1.9 ng/L and 0.28 ng/L [4], and the concentration of BPAF in Taihu Lake increased to 140 ng/L two years later [2]. While, few studies have reported the occurrence of BPAF in aquatic environment of other countries with relatively low concentration, for instance, the highest concentrations of BPAF in wastewater of Slovenia and India were 49 ng/L and 1.1 ng/L, respectively [5,6]. Additionally, BPAF was detected in dairy products (0.028 ng/g) and seafood (0.01 ng/g) [7]. Significantly, in human blood, breast milk, and urine, BPAF was detected as high as 17 ng/L, 56 ng/L, and 120 ng/L, correspondingly [[8], [9], [10], [11]]. Meanwhile, BPAF was also found in human body fluids from other countries at μg/L, such as Malaysians and Saudi Arabia [12,13]. Furthermore, compared with BPA, BPAF is more resistant to treatment processes such as photo-degradation and biodegradation [14]. Therefore, identical with BPA, BPAF will be ubiquitous in the environment and may pose a noticeable risk to human health and ecosystem due to its biomagnification and bioaccumulation potential [2].

BPAF can cause adverse effects on early growth and development, embryos/larvae treated by 2 mg/L BPAF for 120 h were all died, and the offspring survival rate was significantly decreased after adult zebrafish exposed to BPAF [15,16]. Furthermore, developmental malformations of zebrafish larvae were observed after BPAF exposure, including cardiac edema, spinal malformation, and craniofacial deformities [17]. As the replacement for BPA, BPAF has also been reported to interrupt the endocrine system of organisms. In previous studies on zebrafish, short-term exposure to BPAF has been proved to induce endocrine disruption of the thyroid by mediating the expression level of genes associated with the hypothalamic-pituitary-thyroidal (HPT) axis, thyroid hormone production, and thyroid gland development, and similar consequence were also found in female mice [18]. According to previous studies, BPAF could disturb physiological processes through estrogen receptors (ERα and/or ERβ) by acting as an agonist or antagonist, and due to substitution with a hydrophobic group, BPAF performs stronger endocrine-disrupting activity than BPA (20 times stronger for ERα and 48 times stronger for ERβ) [19]. Thus, in *in* *vivo* tests, the toxicity of BPAF on aquatic organisms is more severe than that of BPA [20].

Also, BPAF negatively affects the immune system by inhibiting the viability of macrophages and the functions of T-cells [21,22]. Particularly, BPAF induces substantial effects on cytotoxicity, including elevating intracellular reactive oxygen species (ROS), enhancing protein expression of the c-Myc, G protein-coupled receptor (GPER), and Cyclin D1 [23], as well as altering nuclear morphology, cell cycle, and perturbation of cytoskeleton [24]. Thus, many studies have proved that BPAF has diverse toxicity effects, which were either the same with or different from to those of BPA; however, the toxicity mechanisms are still elusive and need further study.

Mitogen-activated protein kinase (MAPK) is a highly conserved module and consists of three different MAPKs: p38 MAPK, extracellular signal-related kinase (ERK), and Jun amino-terminal kinases (JNK) [25]. MAPK signaling pathway plays a crucial role in development, immune response, and hormonal regulation in vertebrate embryogenesis [26]. Several literatures have reported that BPA can modulate neuronal differentiation and inflammatory reaction via MAPK signaling pathway [27,28]. Furthermore, cytoskeleton architecture perturbation and neurotoxicity in *Mus musculus* induced by BPAF exposure are modulated via the activation of MAPK signaling pathway [29,30], and BPAF could also suppress human dendritic cells maturation through the ERK-MAPK signaling pathway which may impair immunoreaction [22]. However, few studies have investigated the relationship between BPAF toxicity and MAPK signaling pathway in bony fishes.

Global transcriptome sequencing (RNA-Seq) is a recently developed technology used to fully explore the underlying mechanism in environmental pollutant-based ecological toxicity on organisms. Thus, we performed RNA-Seq with bioinformatics analyses, including clustering analysis and Kyoto Encyclopedia of Genes and Genomes (KEGG) pathway analysis, to characterize the underlying mechanisms of BPAF toxicity in fish for the first time. After exposure to BPAF, the early development and stress response effects on zebrafish larvae were explored, and the potential of MAPK signaling in these adverse effects was further studied. Our results provide a necessary understanding for determining the environmental and ecological risks of BPAF at environmentally related concentrations.

## 2 Materials and methods

### 2.1 Chemicals and reagents

BPAF (>98%, CAS 1478-61-1) and dimethyl sulfoxide (DMSO, >99%, CAS 67-68-5) were provided by Aladdin (Shanghai, China). Stock solution (10 g/L) was obtained by dissolving BPAF powder in DMSO and stored at 4 °C. The fresh stock solution was made in each experiment. Other chemicals utilized were of analytic grade and obtained from Aladdin (Shanghai, China).

### 2.2 Zebrafish husbandry

All procedures were approved by the Health Science Animal Care and Use Committee at Southern University of Science and Technology (JY2019067). Adult AB strain wild-type zebrafish (*Danio rerio*) were maintained in a recirculated water farming system at 28 ± 0.5 °C with a light-to-dark cycle of 14: 10 h and fed with live brine shrimp twice per day. Two male and two female fish were paired in breeding tanks overnight before spawning, and the fertilized embryos, examined under a stereomicroscope, were collected in the following morning. Test solutions were made in E3 medium as described by Qiu et al. [31]. The embryos were kept in the test solutions within a light incubator with a switchable light-to-dark photoperiod of 14: 10 h at 28 ± 0.5 °C.

### 2.3 Experimental design

Zebrafish embryos were mixed at 4 h post-fertilization (hpf) and randomly distributed in 90 mm culture dishes containing 25 mL of exposure solution at a concentration of 50 zebrafish embryos per culture dish. The exposure concentrations of BPAF were set up at 0.1, 1, 10, and 100 μg/L. A blank control (E3) and a solvent control (0.001% DMSO in E3) were designed in parallel with the BPAF exposure groups, and three replicates of 50 embryos were used for each treated group. The total treatment solution was replaced daily. Also, the mortality and hatching status of embryos were checked daily. Body length and malformations of zebrafish larvae were measured and documented under a stereomicroscope. After 120 h of exposure, zebrafish larvae of each exposure group were collected and then stored at −80 °C for RNA isolation and biochemical assays.

### 2.4 RNA isolation, cDNA library construction, and sequencing

Total RNA was extracted from larvae after exposure to DMSO and BPAF at four different concentrations using TRIzol® Reagent (Invitrogen, USA). The NanoDrop spectrophotometer and an Agilent 2100 bioanalyzer (Thermo Fisher, MA, USA) were employed to qualify and quantify RNA of each sample. Three replicates were pooled as one treatment sample, and two independent treatments for each group were used for RNA-Seq analysis (n = 2). Premium RNA samples were selected to construct a cDNA library with commercial kits (Mega Genomics, Beijing, China), and then paired-end read sequencing was performed with BGISEQ500 platform (Shenzhen, China).

### 2.5 Bioinformatics analyses

After obtained the raw data of ten samples, the quality control and alignment process was performed with the method described in the study by Qiu et al. [32], and then obtained all expressed genes in zebrafish larvae. The fragments per kilobase of the transcript, per million mapped reads (FPKM) values of the expressed genes were calculated using a previous method [33]. Differences expression analysis between DMSO group and each BPAF-treated group were assessed using DEGseq. Genes with |log_2_(fold-change)| larger than 1 and adjust *p*-value lower than 0.001 were screened as significantly differentially expressed genes (DEGs). A minus-versus-add (MA) plot and a hierarchical cluster analysis of DEGs were performed with the packages ggplot2 and pheatmap in R, respectively. The Venn diagram of the four DEGs datasets was then analyzed.

Dose-response, occurring at the cell, tissue, and organism levels in response to nutrients, pharmacological compounds, and endocrine disrupting chemicals, provides important information to researchers and risk assessors alike [34]. Noise-robust soft clustering was carried out for samples in the four BPAF-treated groups and the DMSO group employing the R package Mfuzz. According to the changes in gene expression at five different dose points, genes were clustered into 12 different groups. Two clusters with up- and down-regulation genes were selected and gene enrichment analysis of these genes was performed with BiNGO plugin in Cytoscape software [35]. The Gene Ontology (GO) terms in different biological processes and the significantly enriched KEGG pathway for the two clusters and DEGs were identified using the phyper function in R. Moreover, a gene set was considered enriched significantly basing on the threshold (*p* < 0.05, false discovery rate [FDR] < 0.01). The protein-protein interaction (PPI) network of the mitogen-activated protein kinase (MAPK) signaling pathway was performed from the STRING database and visualized by Cytoscape 3.6.1 with the method described in our previous study [32]. The plugin *cytoHubba* [36] from Cytoscape was employed to identify crucial hub genes in PPI network by five algorithms: Maximal Clique Centrality (MCC), Density of Maximum Neighborhood Component (DMNC), Edge Percolated Component (EPC), Closeness, and Degree.

### 2.6 Quantitative real-time PCR (qRT-PCR) analysis

Total RNA samples isolated from DMSO and the four BPAF exposure groups were used for qRT-PCR to detect the mRNA level of target genes. cDNA (1 μg) of each sample was synthesized following the protocols of QuantScript RT Kits (Tiangen Bio, Beijing, China). Five hub genes consisting of MYC proto-oncogene, bHLH transcription factor a (*myca*), fibroblast growth factor 3 (*fgf3*), caspase 3, apoptosis-related cysteine peptidase b (*casp3b*), mitogen-activated protein kinase kinase 1 (*map2k1*), and fibroblast growth factor 4 (*fgf4*), as well as 11 randomly selected genes, including growth factor receptor-bound protein 2b (*grb2b*), mitogen-activated protein kinase 12a (*mapk12a*), arrestin, beta 2a (*arrβ2a*), TEK tyrosine kinase, endothelial (*tek*), tumor protein p53 (*tp53*), colony stimulating factor 1 receptor, b (*csf1rb*), fibroblast growth factor 10a (*fgf10a*), fms related receptor tyrosine kinase 4 (*flt4*), epidermal growth factor receptor-like (*LOC571431*), vascular endothelial growth factor Ab (*vegfab*), and MYC proto-oncogene, bHLH transcription factor b (*mycb*), in the PPI network were validated by qRT-PCR. The reaction mixture of qRT-PCR in a 20-μL volume was performed with the FastStart Universal SYBR Green Master kit (Roche, Switzerland) and monitored by CFX Connect Real-Time PCR Detection System (Bio-Rad, USA). The relative expression levels of target genes were evaluated employing the 2^–△△Ct^ method normalized with respect to that of the reference gene Ribosomal protein L13A (*rpl13a*) [37]. Three biological replicates performed in all experiments. The primer sequences of *rpl13a* and selected genes were designed using Primer Premier 6.0 (Table S1).

### 2.7 Detection of oxidative stress indexes and immune-related parameter

The activity of superoxide dismutase (SOD), total antioxidant capacity (T-AOC), and lipid peroxidation (LPO) level were evaluated at 120 hpf by water-soluble tetrazolium-1 (WST-1), 2,2′-azinobis(3-ethylbenzthiazoline-6-sulphonate (ABTS), and thiobarbituric acid (TBA) methods, respectively (Nanjing Jiancheng Bioengineering Institute, China). LPO, the oxidative parameter, was determined by the malondialdehyde (MDA) level. Moreover, the Alkaline phosphatase (AKP) activity was determined to employ disodium phenyl phosphate-4-aminoantipyrine-potassium ferricyanide to indicate the effects of BPAF on immunoregulation in zebrafish larvae. The kit obtained from Nanjing Jiancheng Bioengineering Institute (China) and a fluorescence microplate reader (Thermo Fisher Scientific, USA) were used to test the AKP activity.

### 2.8 Statistical analysis

Data were analyzed with SPSS Statistics 22 (USA) and presented as means ± standard deviation (SD) in this study. One-way analysis of variance (ANOVA) with least significant difference (LSD) test was carried out to evaluate the intergroup differences. Significant differences between DMSO- and BPAF-treated groups are displayed in figures by asterisks (∗, *p* < 0.05). The figures were drawn by Cytoscape 3.6.1 and OriginPro 9.1 (USA).

## 3 Results

### 3.1 Effects of BPAF on development, morphology, oxidative stress, and non-specific immune reactions in zebrafish larvae

Zebrafish embryos were treated by varying concentrations of BPAF (0.1, 1, 10, and 100 μg/L) to test the effects of BPAF on fish development. The hatching rate of zebrafish embryos significantly reduced after exposure to environmental relevant concentrations (1 and 10 μg/L) of BPAF (Fig. 1a). While body lengths of BPAF-exposed zebrafish larvae didn’t significantly differ from that in DMSO group (Fig. 1b). In addition, the malformation numbers were 1.67 ± 0.58 and 2.33 ± 0.58 in the 10 and 100 μg/L BPAF-treated groups, respectively, relative to 0.00 ± 0.00 in the DMSO group with no significant difference; zebrafish larvae mainly exhibited pericardial edema, scoliosis, and tail malformation (Fig. 1c).

**Fig. 1:**
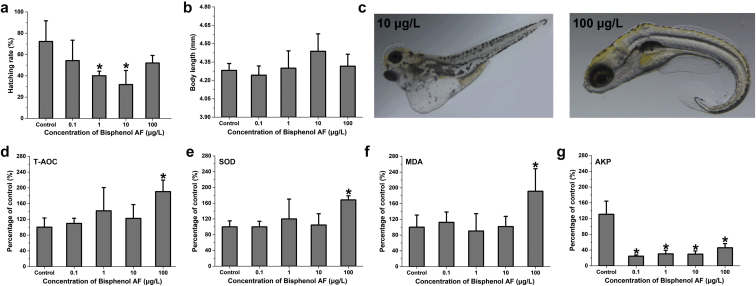
Figure 1(a) Effects of BPAF on hatching rate of zebrafish embryos; (b) Effects of BPAF on body length of zebrafish larvae; (c) Malformations of zebrafish larvae measured under a stereomicroscope in 10 μg/L BPAF (left) and 100 μg/L BPAF (right); (d–g) The change of total antioxidant capacity (T-AOC, d), superoxide dismutase (SOD, e), malondialdehyde level (MDA, f), and alkaline phosphatase (AKP, g) in BPAF-exposed zebrafish larvae for 120 h. Statistical significance was determined by LSD’s test, ∗*p* < 0.05.

The activity of T-AOC and SOD were increased in BPAF-exposed zebrafish larvae, and a significant induction was observed in the 100 μg/L BPAF group (Fig. 1d and e, and Table S2). Moreover, MDA level was significantly induced in the 100 μg/L BPAF-treated group (Fig. 1f and Table S2), and AKP activities were significant reduced in all BPAF-exposed groups (Fig. 1g and Table S2).

### 3.2 The transcriptome changes in BPAF-exposed zebrafish larvae

**Fig. 2:**
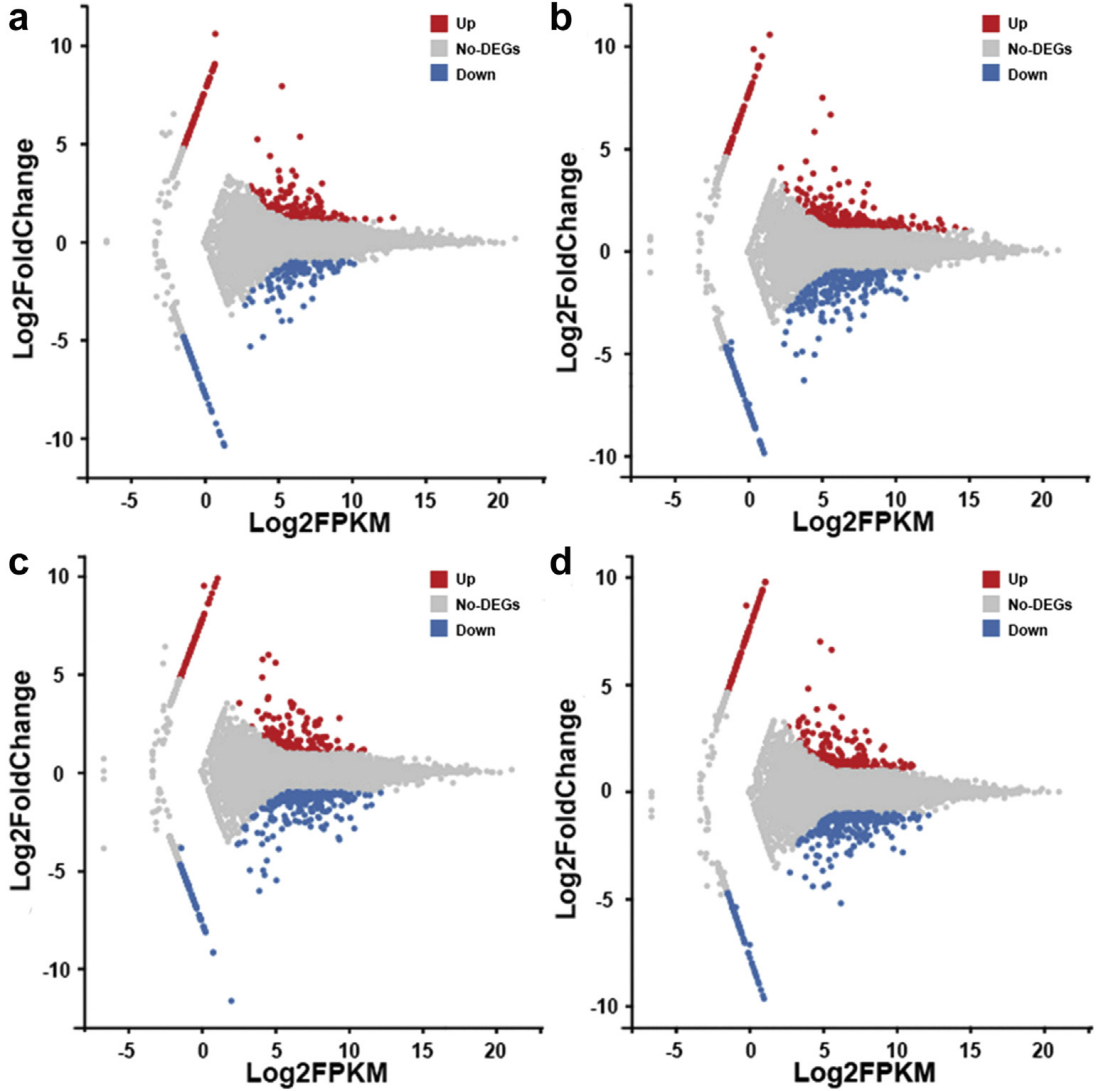
Figure 2The minus-versus-add (MA) plots of differentially expressed genes (DEGs) in 0.1 μg/L (a), 1 μg/L (b), 10 μg/L (c), and 100 μg/L (d) bisphenol AF (BPAF) exposure groups compared to the DMSO group. In MA plots, red and blue dots represent up-regulation and down-regulation DEGs, respectively, and gray dots represent the non-significant changed genes. The Y-axis and X-axis are log2 fold changes and log2 FPKM of all genes, respectively.

**Fig. 3:**
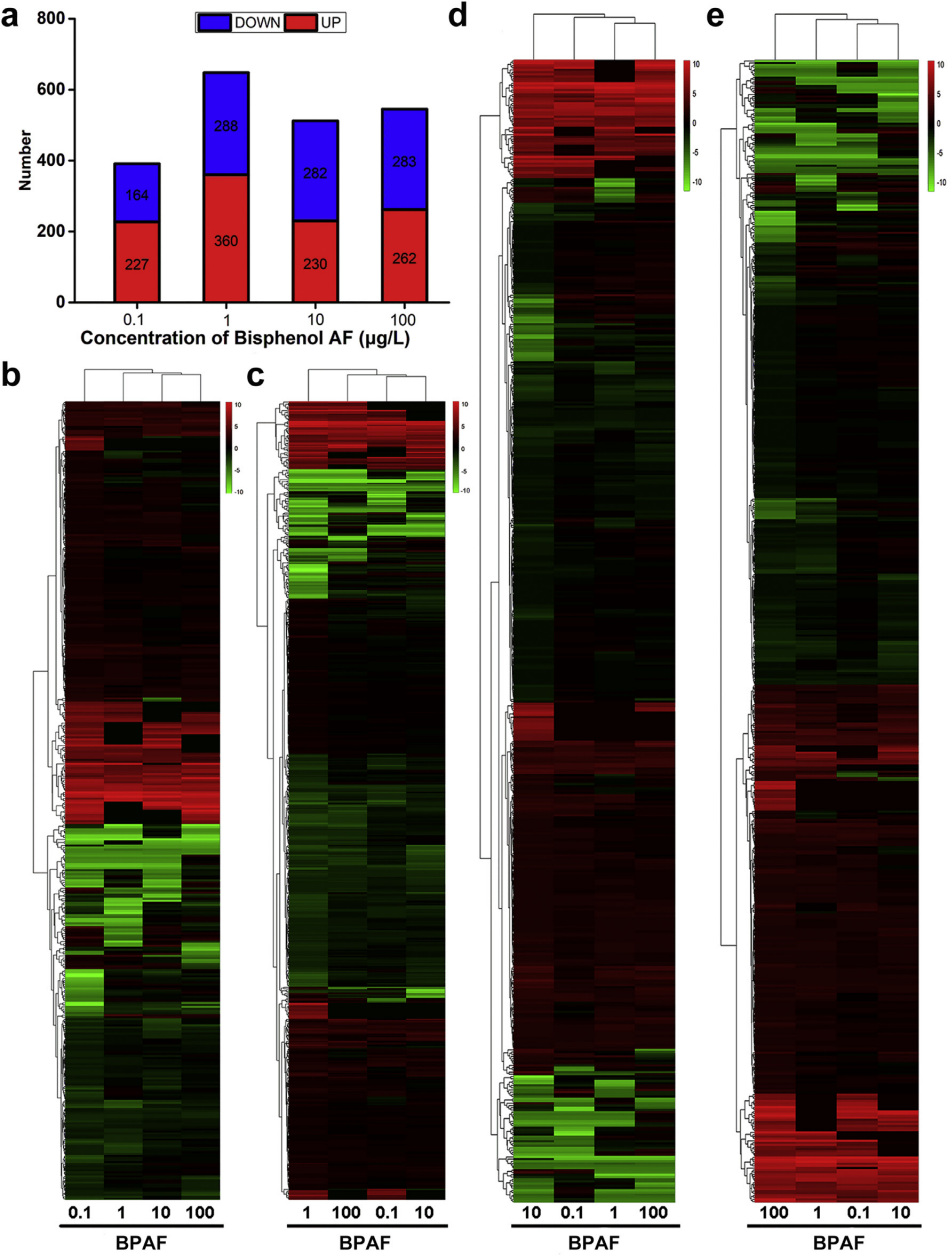
Figure 3(a) Quantities of DEGs in the four BPAF exposure groups relative to the DMSO group; (b–e) Hierarchical clustering analysis of the gene expression patterns of each DEG obtained from the four BPAF groups at 0.1 μg/L (b), 1 μg/L (c), 10 μg/L (d), and 100 μg/L (e), in accordance with their gene expression level in the other three treatment groups. The colors going from red (up-regulation) to green (down-regulation) on the codes indicate the expression change (log2 fold change) in the BPAF-treated groups compared with the DMSO group.

We obtained about 139.1, 141.1, 132.4, 140.6, and 138.1 million clean reads from 0.1, 1, 10, 100 μg/L BPAF and DMSO groups, respectively. The Q20 values of each sample were all above 97.8%, and the mapped ratios in ten samples were from 77.20% to 78.62% (Table S3). Then, the DEGs in each comparison were analyzed, and the MA plot (Fig. 2) showed the expression change of the genes compared between the four BPAF treatment groups and the DMSO group. 391 (227 up- and 164 down-regulated), 648 (360 up- and 288 down-regulated), 512 (230 up- and 282 down-regulated), and 545 (262 up- and 283 down-regulated) DEGs in zebrafish larvae were obtained after exposure to 0.1, 1, 10, and 100 μg/L BPAF, correspondingly (Fig. 3a). Furthermore, Fig. 3b–e showed the hierarchical clustering results of each DEG set in the four BPAF-treated groups, and similar expression patterns in different concentrations of BPAF were obtained.

### 3.3 Clustering analyses and the identification of key functions

**Fig. 4:**
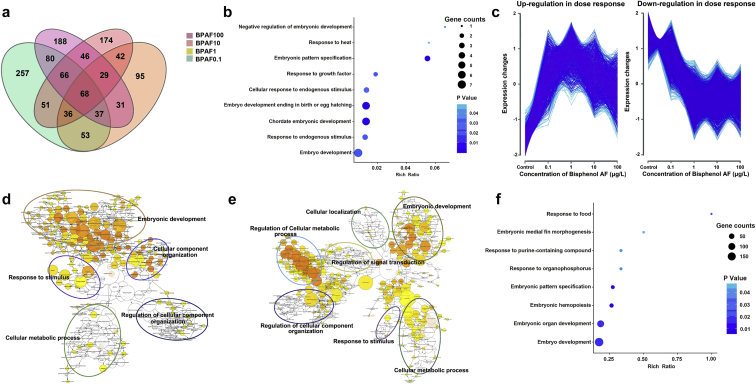
Figure 4(a) The Venn diagram of all obtained DEGs between the four BPAF treatment groups compared to the DMSO group; (b) Gene Ontology (GO) enrichment analysis of the 68 common DEGs. The Y-axis presents GO terms, X-axis presents the proportion of candidate genes among total genes in each term, circle sizes indicate the number of DEGs, and deeper color indicates lower *p* value; (c) The dose-response of up-regulation and down-regulation genes in the four BPAF exposure groups compared with the DMSO group; (d and e) GO enrichment analysis in biological networks of the up-regulation genes (d) and down-regulation genes (e). The nodes represent the GO terms, colors indicate the GO terms enriched significantly (*p* < 0.05), and node sizes represent the numbers of genes in each GO term; (f) GO enrichment analysis of the dose-response genes.

Among all the DEG datasets obtained above, we further investigated how these four different concentrations of BPAF affected the same set of genes, and 68 common DEGs were obtained according to the Venn diagram (Fig. 4a and Table S4). GO enrichment analysis of these 68 common DEGs was conducted and nine significantly enriched GO terms were obtained (Fig. 4b), including embryo development (embryo development ending in birth or egg hatching, embryonic pattern specification, and negative regulation of embryonic development, etc.) and stimulus-response (response to endogenous stimulus, response to heat, and response to growth factor, etc.).

In order to explore the dose-response of BPAF on zebrafish larvae, the clustering analysis of all expressed genes was further studied. As shown in Fig. 4c, two similar expression patterns of the BPAF exposure groups and the DMSO group, including up-regulation in dose-response (cluster1, left) with 1613 genes and down-regulation in dose response (cluster2, right) with 2488 genes, were obtained by clustering analysis. GO functions of the two clusters were further identified, and the major networks in cluster1 were embryonic development, response to stimulus, cellular component organization, and cellular metabolic process (Fig. 4d). For cluster2, embryonic development, response to stimulus, regulation of signal transduction, and regulation of cellular metabolic process were the main functional networks (Fig. 4e). In addition, eight significantly enriched GO terms were obtained with the two gene clusters, and interestingly, these eight GO terms were also divided into two main parts (Fig. 4f), including embryo development (embryonic organ development, embryonic hemopoiesis, and embryonic pattern specification, etc.) and stimulus-response (response to organophosphorus, response to food, and response to purine-containing compound, etc.), which were similar with those of the GO terms from the 68 common genes.

### 3.4 Identification of key pathways with KEGG and hub genes associated with BPAF treatments

**Fig. 5:**
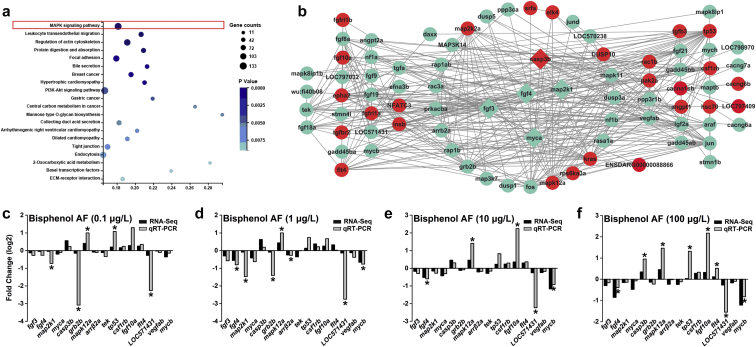
Figure 5(a) The significantly enriched Kyoto Encyclopedia of Genes and Genomes (KEGG) pathways of two gene clusters; (b) Protein-protein interaction (PPI) network of hub genes with their interacted genes in MAPK signaling pathway, red and green correspond to up- and down-regulated genes. Rhombuses indicate hub genes and circle indicate non-hub genes. (c–f) Comparison of the mRNA expression changes (log2 fold change) of five hub genes (*fgf3*, *fgf4*, *map2k1*, *myca*, and *casp3b*) and 11 randomly selected genes (*grb2b*, *mapk12a*, *arrβ2a*, *tek*, *tp53*, *csf1rb*, *fgf10a*, *flt4*, *LOC571431*, *vegfab*, and *mycb*) in the PPI network of MAPK signaling pathway between the results of qRT-PCR and RNA -Seq for BPAF in 0.1 μg/L (c), 1 μg/L (d), 10 μg/L (e), and 100 μg/L (f) exposure groups, relative to the DMSO group. Statistical significance was determined by LSD’s test, ∗*p* < 0.05.

To further examine the function of the two gene clusters in the dose-response analysis, KEGG pathway analyses were employed and MAPK signaling pathway, which included 126 genes, was highly enriched (*p* = 0.0001, *adjp* = 0.0095) (Fig. 5a and Table S5). To explore the interaction of these 126 genes in MAPK signaling pathway, the PPI network was then analyzed with the STRING database and presented with Cytoscape, comprising 81 nodes and 356 edges (Fig. 5b), in which, a total of five hub genes, including *fgf3*, *fgf4*, *map2k1*, *myca*, and *casp3b*, were screened by the overlap of the top 22 genes from five calculation methods in *cytoHubba* (Fig. S1).

Moreover, to validate these results, qRT-PCR analysis was performed on the five hub genes and 11 randomly selected genes in the PPI network, including six up-regulated genes (*casp3b*, *mapk12a*, *tp53*, *csf1rb*, *fgf10a*, and *flt4*) and 10 down-regulated genes (*fgf3*, *fgf4*, *map2k1*, *myca*, *grb2b*, *arrβ2a*, *tek*, *LOC571431*, *vegfab*, and *mycb*) in all BPAF-treated groups (Fig. 5c–f and Table S6). The expression levels of target genes measured by the qRT-PCR were comparatively consistent with those in RNA-seq. Among all the hub genes, gene *casp3b* was induced significantly in the 100 μg/L BPAF-exposed group, while *map2k1* was decreased significantly in the 0.1 and 1 μg/L BPAF-exposed groups. Meanwhile, *fgf4* expression was reduced significantly in all BPAF treatment group except for 0.1 μg/L.

## 4 Discussion

Combining the results of the omics and morphological analyses, our study demonstrated that exposure to BPAF could significantly affect embryo development and stress response of zebrafish larvae. As an important BPA substitutes, BPAF has been proved to have the higher binding potentials with ER than BPA, and the estrogenicity and lethality to zebrafish larvae were larger than BPA [17,38], thus, to explore the underlying mechanism on the adverse effect of embryo development and stress response in BPAF-exposed zebrafish larvae was meaningful to evaluate the similar or different toxicity between BPAF and BPA.

The MAPK signaling pathway, identified by KEGG analysis, is important in development, immune response, and hormonal regulation in vertebrate embryogenesis [26]. Previous studies have reported that bisphenols can modulate neuronal differentiation and cause neurotoxicity via MAPK signaling pathway [27,39]. Further, the inflammatory reaction mediated by BPA was also partially administered with MAPK signaling pathway [28]. Similar to these studies, our KEGG analysis results also showed that MAPK signaling pathway was significantly enriched by genes in dose-response following BPAF treatment, and the gene expression changes of mRNAs in this pathway were validated by qRT-PCR, further indicating that the toxicity of BPAF on zebrafish larvae was mainly mediated by MAPK signaling pathway.

The PPI analysis results showed that five hub genes (*myca*, *fgf3*, *fgf4*, *map2k1*, and *casp3b*) were identified to be the core nodes linking MAPK signaling pathway. The gene *myca* is a downstream transcription factor in MAPK/ERK signaling pathway which plays a critical role during early development; the knockdown of *myca* in mice embryos leads to developmental arrest at early cleavage stages [40,41]. Thus, reduction of the *myca* level may mediate early developmental characteristics such as hatchability of zebrafish embryos. Activated by fibroblast growth factors (FGFs), ERK can regulate the formation of tissues or organs such as limbs, brains, somites, eyes, and inner ears in vertebrates [42]. The genes *fgf3* and *fgf4* are two important members in FGF family, and *fgf3* is mainly related to the development of otic features as well as hindbrain and forebrain in zebrafish [43,44]. Moreover, the loss of *fgf4* expression during late gastrulation could result in abnormal morphology of the thoracic vertebrae and ribs, malformation of lumbar and sacral vertebrae, and absence of tail vertebrae [45]. Thus, the down-regulation of *fgf3* and *fgf4* by BPAF may lead to developmental deficits and malformation in zebrafish larvae [46,47]. ERK is phosphorylated and activated by mitogen-activated protein kinase (MEK) consisting of MEK1 (*map2k1*) and MEK2 (*map2k2*) to produce phosphorylated ERK (pERK) [48]. The activation of MEK1/2-ERK1/2 signal pathway could mediate inflammatory cytokines production to induce inflammatory responses [49,50]. Furthermore, MEK1/2 was mainly expressed in systemic immune organs, especially the head kidney of fish [51]. In this study, mRNA of *map2k1* was reduced by BPAF exposure, which implied that immune function was inhibited in zebrafish larvae through the suppression of the MEK1/2-ERK1/2 signal pathway. In zebrafish, JNK signal pathway, which is sensitive to cellular stresses or extracellular signals, is related to organogenesis, embryonic development, and immune responses [52,53]. Caspase-3 (*casp3b*) is the executioner of apoptosis in JNK [54], and oxidative stress triggered by ROS is involved in the process of apoptotic occurrence [55]. BPA or its analog bisphenol F could activate *caspase 3* with the mediation of the MAPK/JNK signaling pathway to trigger apoptosis in cells [56,57]. Hence, similar to these studies, the induction in *caspase 3* expression of the BPAF-treated groups may have also contributed to the abnormal immune response of zebrafish larvae.

Based on the analysis of hub genes, *myca*, *fgf3*, and *fgf4* were mainly related to early development, and *map2k1* and *casp3b* were mainly related to oxidative stress. Moreover, GO enrichment analysis of 68 common DEGs in different concentrations of BPAF was significantly enriched at the GO terms of embryo development and stimulus-response. This may indicate that the interaction of hub genes and other genes in MAPK signaling pathway are able to mediate the early development and stress response induced by BPAF, which we obtained from GO analysis of DEGs and the cluster genes in RNA-Seq. A previous study showed that the MAPK gene family is important in the process of zebrafish embryogenesis [58], and the MAPK signal could also mediate oxidative stress-induced apoptosis [59]. In the prior studies on BPA, Liu et all [60]. revealed that BPA could inhibit cells proliferation in embryonic midbrain of rats with a decrease in phosphorylation levels of JNK; in addition, BPA could suppress the proliferation of other cells through MAPK signaling pathway [61]. Furthermore, in rat brains, ROS production induced by BPA was related to the regulation of the MAPK signal [62], and BPA could also affect the SOD level by activating the ERK signal [63]. These prior studies and our obtained results suggest that the effects of BPAF on early development and stress response might be mediated by MAPK signaling pathway.

In the present study, both environmental concentration (10 μg/L) and higher concentration (100 μg/L) of BPAF treatment cause the malformation of some zebrafish larvae, suggesting the early growth of zebrafish was affected, which was consistent with the GO analysis results in RNA-Seq. In line with the present study, other bisphenols including BPA, bisphenol F, and bisphenol S were also proved to affect the development of zebrafish [17,64,65]. In addition, previous studies have revealed that bisphenols were able to induce the oxidative stress [31,66], and similarly, BPAF could also result in the induction of ROS in human and mouse cells [39,67]. We found that only 100 μg/L BPAF exposure could increase the content of oxidative stress indexes, T-AOC, SOD, and MDA, in zebrafish larvae. However, AKP, an important enzyme involved in the immune response of cell and displays the defense capability of body to against exogenous microbial infection [68], was significantly reduced in all BPAF treatment groups. These results indicated the oxidative stress induction and the immune-response interference in the early stages of zebrafish, which is in line with the findings in recent studies [31,69]. Consequently, this further verified the results obtained by GO analysis that the stimulus-response of zebrafish larvae was disturbed by BPAF, and this may mediate by MAPK signaling pathway described above.

Interestingly, the hatching rate of zebrafish embryos in environmental relevant concentration (1 and 10 μg/L) of BPAF treatment groups were significantly decreased, which is consistent with previous studies [38]. Moreover, among all the DEGs in low concentration BPAF treatment groups, two important genes hairy-related 6 (*her6*) and anthrax toxin receptor 2a (*antxr2a*), controls the somitogenesis and morphogenesis of zebrafish [70,71], were significantly reduced by BPAF, this may account for the delay of growth and hatching. As for oxidative stress indexes, T-AOC, SOD, and MDA level were only slightly increased in low concentration BPAF treatment groups with no significance. However, two other DEGs, progestin and adipoQ receptor family member IIIa (*paqr3a*), an enzyme in the Golgi that is important in maintaining cellular cholesterol homeostasis [72], and autocrine motility factor receptor b (*amfrb*), highly expressed in liver and brain of zebrafish to regulate hepatic endoplasmic reticulum stress and lipid metabolism [73], were significantly induced by 0.1, 1, and 10 μg/L BPAF, leading the high concentration of cholesterol and fatty acid to induce the production of cellular ROS in zebrafish [74]. Thus, the process of early growth and stimulus-response in zebrafish larvae were also affected by low concentration BPAF treatment.

Thus, from the discussion above, we have built the relationship between the toxicity effects and MAPK signaling in zebrafish larvae upon BPAF treatment, which was reflected by the results of morphology and pathology. However, the specific role of this pathway needs to be further demonstrated by analyzing corresponding protein expression, including p38, JNK, and ERK.

## 5 Conclusions

In this study, we explored the toxicity of BPAF on the early stages of zebrafish at environmentally relevant concentrations, and numerous genes in zebrafish larvae were significantly dysregulated by BPAF treatment. The early growth and stimulus-response of zebrafish larvae were inhibited in BPAF-exposed zebrafish larvae, and this may be induced by the disruption of MAPK signaling pathway between the interactions of hub genes with other genes. Moreover, the oxidative stress indexes and AKP levels were impaired, indicating the oxidative stress induction and immune-response interference in zebrafish larvae after the BPAF treatments. This suggests that intensive studies of the BPAF toxicity to the immune system are needed to be conducted.

## Declaration of interests

The authors declare that they have no known competing financial interests or personal relationships that could have appeared to influence the work reported in this paper.

## Figures and Tables

**figs1:**
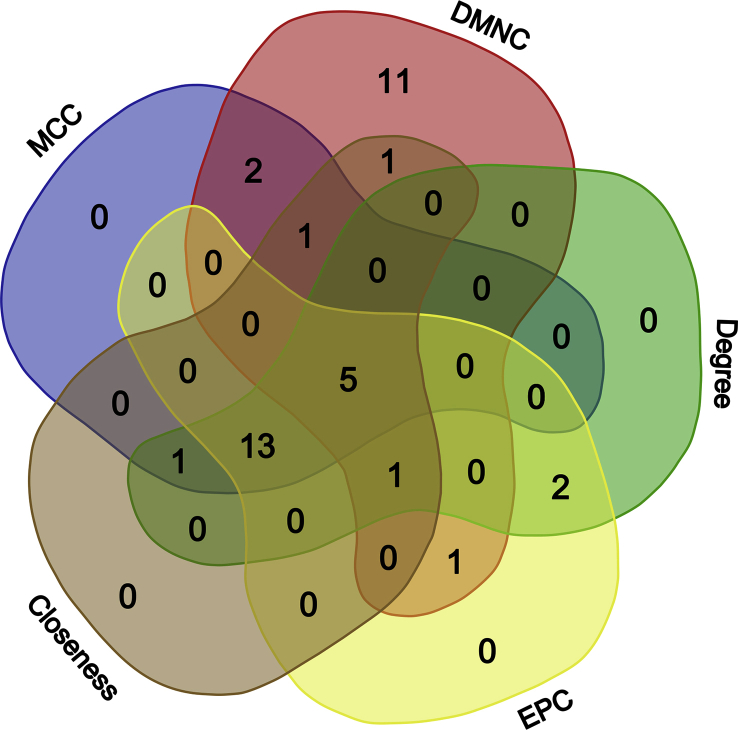


## References

[1] Feng Y.X., Yin J., Jiao Z.H., Shi J.C., Li M., Shao B. (2012). Bisphenol AF may cause testosterone reduction by directly affecting testis function in adult male rats. Toxicol. Lett..

[2] Wang Q., Chen M., Shan G.Q., Chen P.Y., Cui S., Yi S.J., Zhu L.Y. (2017). Bioaccumulation and biomagnification of emerging bisphenol analogues in aquatic organisms from Taihu Lake, China. Sci. Total Environ..

[3] Song S.J., Ruan T., Wang T., Liu R.Z., Jiang G.B. (2012). Distribution and preliminary exposure assessment of bisphenol AF (BPAF) in various environmental matrices around a manufacturing plant in China. Environ. Sci. Technol..

[4] Jin H.B., Zhu L.Y. (2016). Occurrence and partitioning of bisphenol analogues in water and sediment from Liaohe River basin and Taihu Lake, China. Water Res..

[5] Cesen M., Lenarcic K., Mislej V., Levstek M., Kovacic A., Cimrmancic B., Uranjek N., Kosjek T., Heath D., Dolenc M.S., Heath E. (2018). The occurrence and source identification of bisphenol compounds in wastewaters. Sci. Total Environ..

[6] Karthikraj R., Kannan K. (2017). Mass loading and removal of benzotriazoles, benzothiazoles, benzophenones, and bisphenols in Indian sewage treatment plants. Chemosphere.

[7] Liao C.Y., Kannan K. (2013). Concentrations and profiles of bisphenol A and other bisphenol analogues in foodstuffs from the United States and their implications for human exposure. J. Agric. Food Chem..

[8] Niu Y.M., Wang B., Zhao Y.F., Zhang J., Shao B. (2017). Highly sensitive and high-throughput method for the analysis of bisphenol analogues and their halogenated derivatives in breast milk. J. Agric. Food Chem..

[9] Jin H.B., Zhu J., Chen Z.J., Hong Y.J., Cai Z.W. (2018). Occurrence and partitioning of bisphenol analogues in adults’ blood from China. Environ. Sci. Technol..

[10] Song S.M., Duan Y.S., Zhang T., Zhang B., Zhao Z., Bai X.Y., Xie L., He Y., Ouyang J.P., Huang X.F., Sun H.W. (2019). Serum concentrations of bisphenol A and its alternatives in elderly population living around e-waste recycling facilities in China: associations with fasting blood glucose, Ecotoxical. Environ. Saf..

[11] Zhang B., He Y., Zhu H., Huang X., Bai X., Kannan K., Zhang T. (2020). Concentrations of bisphenol A and its alternatives in paired maternal-fetal urine, serum and amniotic fluid from an e-waste dismantling area in China. Environ. Int..

[12] Wiraagni I.A., Mohd M.A., Rashid R.A., Haron D. (2020). Trace level detection of bisphenol A analogues and parabens by LC-MS/MS in human plasma from Malaysians. BioMed Res. Int..

[13] Asimakopoulos A.G., Xue J., De Carvalho B.P., Iyer A., Abualnaja K.O., Yaghmoor S.S., Kumosani T.A., Kannan K. (2016). Urinary biomarkers of exposure to 57 xenobiotics and its association with oxidative stress in a population in Jeddah, Saudi Arabia. Environ. Res..

[14] Choi Y. (2016).

[15] Song M.Y., Liang D., Liang Y., Chen M.J., Wang F.B., Wang H.L., Jiang G.B. (2014). Assessing developmental toxicity and estrogenic activity of halogenated bisphenol A on zebrafish (*Danio rerio*). Chemosphere.

[16] Shi J.C., Jiao Z.H., Zheng S., Li M., Zhang J., Feng Y.X., Yin J., Shao B. (2015). Long-term effects of Bisphenol AF (BPAF) on hormonal balance and genes of hypothalamus-pituitary-gonad axis and liver of zebrafish (*Danio rerio*), and the impact on offspring. Chemosphere.

[17] Moreman J., Lee O., Trznadel M., David A., Kudoh T., Tyler C.R. (2017). Acute toxicity, teratogenic, and estrogenic effects of bisphenol A and its alternative replacements bisphenol S, bisphenol F, and bisphenol AF in zebrafish embryo-larvae. Environ. Sci. Technol..

[18] Meng Z.Y., Wang D.Z., Yan S., Li R.S., Yan J., Teng M.M., Zhou Z.Q., Zhu W.T. (2018). Effects of perinatal exposure to BPA and its alternatives (BPS, BPF and BPAF) on hepatic lipid and glucose homeostasis in female mice adolescent offspring. Chemosphere.

[19] Matsushima A., Liu X.H., Okada H., Shimohigashi M., Shimohigashi Y. (2010). Bisphenol AF is a full agonist for the estrogen receptor ER alpha but a highly specific antagonist for ER beta. Environ. Health Perspect..

[20] Tisler T., Krel A., Gerzelj U., Erjavec B., Dolenc M.S., Pintar A. (2016). Hazard identification and risk characterization of bisphenols A, F and AF to aquatic organisms. Environ. Pollut..

[21] Chen Y., Xu H.S., Guo T.L. (2018). Modulation of cytokine/chemokine production in human macrophages by bisphenol A: a comparison to analogues and interactions with genistein. J. Immunot..

[22] Svajger U., Dolenc M.S., Jeras M. (2016). *In vitro* impact of bisphenols BPA, BPF, BPAF and 17beta-estradiol (E2) on human monocyte-derived dendritic cell generation, maturation and function. Int. Immunopharm..

[23] Lei B.L., Sun S., Zhang X.L., Feng C.L., Xu J., Wen Y., Huang Y.G., Wu M.H., Yu Y.X. (2019). Bisphenol AF exerts estrogenic activity in MCF-7 cells through activation of Erk and PI3K/Akt signals via GPER signaling pathway. Chemosphere.

[24] Liang S.X., Yin L., Yu K.S., Hofmann M.C., Yu X.Z. (2017). High-content analysis provides mechanistic insights into the testicular toxicity of bisphenol A and selected analogues in mouse spermatogonial cells. Toxicol. Sci..

[25] Zhang W., Liu H.T. (2002). MAPK signal pathways in the regulation of cell proliferation in mammalian cells. Cell Res..

[26] Yan L., Carr J., Ashby P.R., Murry-Tait V., Thompson C., Arthur J.S. (2003). Knockout of ERK5 causes multiple defects in placental and embryonic development. BMC Dev. Biol..

[27] Seki S., Aoki M., Hosokawa T., Saito T., Masuma R., Komori M., Kurasaki M. (2011). Bisphenol-A suppresses neurite extension due to inhibition of phosphorylation of mitogen-activated protein kinase in PC12 cells. Chem. Biol. Interact..

[28] Zhu J., Jiang L., Liu Y., Qian W., Liu J., Zhou J., Gao R., Xiao H., Wang J. (2015). MAPK and NF-kappaB pathways are involved in bisphenol A-induced TNF-alpha and IL-6 production in BV2 microglial cells. Inflammation.

[29] Wu D., Huang C.J., Jiao X.F., Ding Z.M., Zhang S.X., Miao Y.L., Huo L.J. (2019). Bisphenol AF compromises blood-testis barrier integrity and sperm quality in mice. Chemosphere.

[30] Lee S., Kim Y.K., Shin T.Y., Kim S.H. (2013). Neurotoxic effects of bisphenol AF on calcium-induced ROS and MAPKs. Neurotox. Res..

[31] Qiu W.H., Shao H.Y., Lei P.H., Zheng C.M., Qiu C.X., Yang M., Zheng Y. (2018). Immunotoxicity of bisphenol S and F are similar to that of bisphenol A during zebrafish early development. Chemosphere.

[32] Qiu W.H., Liu X.J., Yang F., Li R.Z., Xiong Y., Fu C.X., Li G.R., Liu S., Zheng C.M. (2020). Single and joint toxic effects of four antibiotics on some metabolic pathways of zebrafish (*Danio rerio*) larvae. Sci. Total Environ..

[33] Trapnell C., Williams B.A., Pertea G., Mortazavi A., Kwan G., van Baren M.J., Salzberg S.L., Wold B.J., Pachter L. (2010). Transcript assembly and quantification by RNA-Seq reveals unannotated transcripts and isoform switching during cell differentiation. Nat. Biotechnol..

[34] Hill C.E., Myers J.P., Vandenberg L.N. (2018). Nonmonotonic dose-response curves occur in dose ranges that are relevant to regulatory decision-making. Dose-Response.

[35] Shannon P., Markiel A., Ozier O., Baliga N.S., Wang J.T., Ramage D., Amin N., Schwikowski B., Ideker T. (2003). Cytoscape: a software environment for integrated models of biomolecular interaction networks. Genome Res..

[36] Chin C.H., Chen S.H., Wu H.H., Ho C.W., Ko M.T., Lin C.Y. (2014). cytoHubba: identifying hub objects and sub-networks from complex interactome. BMC Syst. Biol..

[37] Tang R.Y., Dodd A., Lai D., McNabb W.C., Love D.R. (2007). Validation of zebrafish (*Danio rerio*) reference genes for quantitative real-time RT-PCR normalization. Acta Biochim. Biophys. Sin..

[38] Mu X.Y., Huang Y., Li X.X., Lei Y.L., Teng M.M., Li X.F., Wang C.J., Li Y.R. (2018). Developmental effects and estrogenicity of bisphenol A alternatives in a zebrafish embryo model. Environ. Sci. Technol..

[39] Lee S., Kim Y.K., Shin T.Y., Kim S.H. (2013). Neurotoxic effects of bisphenol AF on calcium-induced ROS and MAPKs. Neurotox. Res..

[40] Paria B.C., Dey S.K., Andrews G.K. (1992). Antisense C-myc effects on preimplantation mouse embryo development. Proc. Natl. Acad. Sci. U.S.A..

[41] Naz R.K., Kumar G., Minhas B.S. (1994). Expression and role of C-myc protooncogene in murine preimplantation embryonic-development. J. Assist. Reprod. Genet..

[42] Wong K.L., Akiyama R., Bessho Y., Matsui T. (2018). ERK activity dynamics during zebrafish embryonic development. Int. J. Mol. Sci..

[43] Walshe J., Mason I. (2003). Unique and combinatorial functions of Fgf3 and Fgf8 during zebrafish forebrain development. Development.

[44] Leger S., Brand M. (2002). Fgf8 and Fgf3 are required for zebrafish ear placode induction, maintenance and inner ear patterning. Mech. Dev..

[45] Boulet A.M., Capecchi M.R. (2012). Signaling by FGF4 and FGF8 is required for axial elongation of the mouse embryo. Dev. Biol..

[46] Walshe J., Mason I. (2003). Fgf signalling is required for formation of cartilage in the head. Dev. Biol..

[47] Naiche L.A., Holder N., Lewandoski M. (2011). FGF4 and FGF8 comprise the wavefront activity that controls somitogenesis. Proc. Natl. Acad. Sci. U.S.A..

[48] Yao Z., Seger R. (2009). The ERK signaling cascade-Views from different subcellular compartments. Biofactors.

[49] Li D.Q., Luo L.H., Chen Z., Kim H.S., Song X.J., Pflugfelder S.C. (2006). JNK and ERK MAP kinases mediate induction of IL-1 beta, TNF-alpha and IL-8 following hyperosmolar stress in human limbal epithelial cells. Exp. Eye Res..

[50] Jo S.K., Cho W.Y., Sung S.A., Kim H.K., Won N.H. (2005). MEK inhibitor, U0126, attenuates cisplatin-induced renal injury by decreasing inflammation and apoptosis. Kidney Int..

[51] Mo Z.Q., Han R., Wang J.L., Ni L.Y., Su Y.L., Lai X.L., He Z.C., Chen H.P., Li Y.W., Sun H.Y., Luo X.C., Dan X.M. (2019). Characterization and functional analysis of grouper (*Epinephelus coioides*) MEK1 and MEK2. Fish Shellfish Immunol..

[52] Seo J., Asaoka Y., Nagai Y., Hirayama J., Yamasaki T., Namae M., Ohata S., Shimizu N., Negishi T., Kitagawa D., Kondoh H., Furutani-Seiki M., Penninger J.M., Katada T., Nishina H. (2010). Negative regulation of wnt11 expression by jnk signaling during zebrafish gastrulation. J. Cell. Biochem..

[53] Xiao Y.M., Zhou Y.H., Xiong Z., Zou L.J., Jiang M.G., Luo Z.W., Wen S., Liu W.B., Liu S.J., Li W.C. (2013). Involvement of JNK in the embryonic development and organogenesis in zebrafish. Mar. Biotechnol..

[54] Spead O., Verreet T., Donelson C.J., Poulain F.E. (2018). Characterization of the caspase family in zebrafish. PloS One.

[55] Lu Y.J., Luo Q., Cui H.M., Deng H.D., Kuang P., Liu H., Fang J., Zuo Z.C., Deng J.L., Li Y.L., Wang X., Zhao L. (2017). Sodium fluoride causes oxidative stress and apoptosis in the mouse liver. Aging-Us.

[56] Lee S., Suk K., Kim I.K., Jang I.S., Park J.W., Johnson V.J., Kwon T.K., Choi B.J., Kim S.H. (2008). Signaling pathways of bisphenol A-induced apoptosis in hippocampal neuronal cells: role of calcium-induced reactive oxygen species, mitogen-activated protein kinases, and nuclear factor-kappa B. J. Neurosci. Res..

[57] Zhao C., Tang Z., Xie P.S., Lin K.L., Chung A.C.K., Cai Z.W. (2019). Immunotoxic potential of bisphenol F mediated through lipid signaling pathways on macrophages. Environ. Sci. Technol..

[58] Krens S.F., He S., Spaink H.P., Snaar-Jagalska B.E. (2006). Characterization and expression patterns of the MAPK family in zebrafish. Gene Expr. Patterns.

[59] Xu J., Qian J., Xie X., Ma J., Lin L., Fu M., Sun A., Zou Y., Ge J. (2011). Activation of MAPK/ERK1/2 pathways is an important mechanism of oxidative stress-induced apoptosis in mesenchymal stem cells of rats. Heart.

[60] Liu R., Xing L., Kong D., Jiang J., Shang L., Hao W. (2013). Bisphenol A inhibits proliferation and induces apoptosis in micromass cultures of rat embryonic midbrain cells through the JNK, CREB and p53 signaling pathways. Food Chem. Toxicol..

[61] Qu W., Zhao Z., Chen S., Zhang L., Wu D., Chen Z. (2018). Bisphenol A suppresses proliferation and induces apoptosis in colonic epithelial cells through mitochondrial and MAPK/AKT pathways. Life Sci..

[62] Kobayashi K., Liu Y., Ichikawa H., Takemura S., Minamiyama Y. (2020). Effects of bisphenol A on oxidative stress in the rat brain. Antioxidants.

[63] Sauer S.J., Tarpley M., Shah I., Save A.V., Lyerly H.K., Patierno S.R., Williams K.P., Devi G.R. (2017). Bisphenol A activates EGFR and ERK promoting proliferation, tumor spheroid formation and resistance to EGFR pathway inhibition in estrogen receptor-negative inflammatory breast cancer cells. Carcinogenesis.

[64] Yang Q., Yang X., Liu J., Chen Y., Shen S. (2018). Effects of exposure to BPF on development and sexual differentiation during early life stages of zebrafish (*Danio rerio*). Comp. Biochem. Physiol. C Toxicol. Pharmacol..

[65] Duan Z., Zhu L., Zhu L., Kun Y., Zhu X. (2008). Individual and joint toxic effects of pentachlorophenol and bisphenol A on the development of zebrafish (*Danio rerio*) embryo. Ecotoxicol. Environ. Saf..

[66] Pang Q., Li Y., Meng L., Li G., Luo Z., Fan R. (2019). Neurotoxicity of BPA, BPS, and BPB for the hippocampal cell line (HT-22): an implication for the replacement of BPA in plastics. Chemosphere.

[67] Macczak A., Cyrkler M., Bukowska B., Michalowicz J. (2017). Bisphenol A, bisphenol S, bisphenol F and bisphenol AF induce different oxidative stress and damage in human red blood cells (*in vitro* study). Toxicol. *Vitro*.

[68] Li F., Wang H., Liu J., Lin J., Zeng A., Ai W., Wang X., Dahlgren R.A., Wang H. (2016). Immunotoxicity of beta-diketone antibiotic mixtures to zebrafish (*Danio rerio*) by transcriptome analysis. PloS One.

[69] Qiu W.H., Shen Y., Pan C.Y., Liu S., Wu M.H., Yang M., Wang K.J. (2016). The potential immune modulatory effect of chronic bisphenol A exposure on gene regulation in male medaka (*Oryzias latipes*) liver. Ecotoxical. Environ. Saf..

[70] Pasini A., Jiang Y.J., Wilkinson D.G. (2004). Two zebrafish Notch-dependent hairy/Enhancer-of-split-related genes, her6 and her4, are required to maintain the coordination of cyclic gene expression in the presomitic mesoderm. Development.

[71] Castanon I., Abrami L., Holtzer L., Heisenberg C.P., van der Goot F.G., Gonzalez-Gaitan M. (2013). Anthrax toxin receptor 2a controls mitotic spindle positioning. Nat. Cell Biol..

[72] Xu D.Q., Wang Z., Zhang Y.X., Jiang W., Pan Y., Song B.L., Chen Y. (2015). PAQR3 modulates cholesterol homeostasis by anchoring Scap/SREBP complex to the Golgi apparatus. Nat. Commun..

[73] Chen Z.L., Ballar P., Fu Y., Luo J., Du S.J., Fang S.Y. (2014). The E3 ubiquitin ligase gp78 protects against ER stress in zebrafish liver. J. Genet. Genomics.

[74] Wang Y.J., Bian Y., Luo J., Lu M., Xiong Y., Guo S.Y., Yin H.Y., Lin X., Li Q., Chang C.C.Y., Chang T.Y., Li B.L., Song B.L. (2017). Cholesterol and fatty acids regulate cysteine ubiquitylation of ACAT2 through competitive oxidation. Nat. Cell Biol..

