# Supplementary material for: Transcriptomic analysis of bisphenol AF on early growth and development of zebrafish (Danio rerio) larvae
Source: Environ Sci Ecotechnol. 2020 Aug 5;4:100054. doi: 10.1016/j.ese.2020.100054 (PMC9488094; doi:10.1016/j.ese.2020.100054)
Supplement: Multimedia component 1 [file mmc1.docx]

**Table S1.** Primer sequences of selected genes in the present study.

| **Gene name** | **Primers sequence (5’-3’)** | **Accession numbers** |
| --- | --- | --- |
| growth factor receptor-bound protein 2b (*grb2b*) | F: GTCCTCTGGTCCACAATAG  R: TGCCTTCATCTCAATGTAGT | NM_213035.1 |
| mitogen-activated protein kinase 12a (*mapk12a*) | F: TCTGCGGATTCTATTACCAA  R: CATTGCTCTAGCGACTGA | NM_131407.1 |
| arrestin, beta 2a (*arrβ2a*) | F: ACGCCAACTCTCAACAAC  R: CCAGAACTTCCTTATTACTCAC | NM_214681.1 |
| caspase 3, apoptosis-related cysteine peptidase b (*casp3b*) | F: ATTGAGTGTGACGGTGTAG  R: CTGGAAGATTGGATGAGGAT | NM_001048066.2 |
| TEK tyrosine kinase, endothelial (*tek*) | F: GATGGCAATAGAGTCGCTTA  R: CTCGTCGTCACAGTTCAG | NM_131461.1 |
| tumor protein p53 (*tp53*) | F: GGTGAAGGACGAAGGAAG  R: AGTGGAGTGAACAAGAAGAA | NM_001271820.1 |
| colony stimulating factor 1 receptor, b (*csf1rb*) | F: TCCATCAGTGTCTCTTCCA  R: TCTTCCAGCCTCGTTCAT | XM_009295703.3 |
| fibroblast growth factor 10a (*fgf10a*) | F: CCTCGTCGCCTCTTATTC  R: CCTTCTGCTTACCACTGAA | NM_182870.2 |
| fms related receptor tyrosine kinase 4 (*flt4*) | F: CCAGAGCATCCATTCATCA  R: ATACGGCACTAACGAGAAG | NM_130945.2 |
| epidermal growth factor receptor-like (*LOC571431*) | F: ACGGAGATGTGGTGGTTA  R: AGGTGCTGTTCTGCTGTA | XM_695018.9 |
| mitogen-activated protein kinase kinase 1 (*map2k1*) | F: GAGATTAAGCCAGCAATAAGG  R: GCGAGTTGACCAAGATGT | NM_213419.2 |
| fibroblast growth factor 3 (*fgf3*) | F: GGAAACTTTACTGCGCCACA  R: TCCCTTGATTGCCACCACAC | NM_131291.1 |
| fibroblast growth factor 4 (*fgf4*) | F: CCACTCCAACACAACAATG  R: CACAGCACATACATCACAAT | NM_131635.3 |
| vascular endothelial growth factor Ab (*vegfab*) | F: ACCGAACTGACTTGAGAAC  R: CCAACCACTTCACTTCATTC | NM_001328597.1 |
| MYC proto-oncogene, bHLH transcription factor a (*myca*) | F: GGAGGAAGAAGAGGAGGAA  R: CTGTTGATGCTGTGATTGTT | NM_131412.1 |
| MYC proto-oncogene, bHLH transcription factor b (*mycb*) | F: GAGTAGCAGCAGCAGTAG  R: GTAGTTGTGTTGGTGAATGT | NM_200172.1 |

**Table S2.** The fold change of T-AOC, SOD, MDA, and AKP (%) in BPAF treatment groups compared with the control (n = 3).

|  | **T-AOC** | **SOD** | **MDA** | **AKP** |
| --- | --- | --- | --- | --- |
| **0.1 μg/L** | 115.42 | 114.36 | 126.83 | 18.11 |
|  | 94.54 | 86.11 | 81.87 | 16.60 |
|  | 118.69 | 99.39 | 128.11 | 21.26 |
| **1 μg/L** | 198.45 | 167.09 | 137.75 | 29.52 |
|  | 145.42 | 126.05 | 82.52 | 24.11 |
|  | 80.36 | 67.05 | 50.31 | 15.57 |
| **10 μg/L** | 142.24 | 123.16 | 123.37 | 27.52 |
|  | 142.79 | 118.87 | 108.27 | 24.58 |
|  | 82.07 | 71.46 | 72.82 | 15.62 |
| **100 μg/L** | 198.36 | 160.85 | 125.70 | 34.83 |
|  | 157.56 | 125.77 | 229.79 | 27.00 |
|  | 214.51 | 175.71 | 218.52 | 43.16 |

**Table S3.** Raw data quality assessment

| **Sample** | **Raw Reads (M)** | **Clean Reads (M)** | **Q20 (%)** | **Total Mapped (%)** | **Unique Mapped (%)** |
| --- | --- | --- | --- | --- | --- |
| **Control** | 71.86 | 69.04 | 98.48 | 88.02 | 57.8 |
|  | 72.27 | 69.07 | 98.34 | 87.75 | 57.62 |
| **0.1 μg/L** | 72.45 | 69.81 | 98.38 | 88.59 | 58.03 |
|  | 72.04 | 69.28 | 98.38 | 88.16 | 58.08 |
| **1 μg/L** | 74.63 | 71.57 | 98.43 | 87.62 | 57.29 |
|  | 72.17 | 69.58 | 98.4 | 88.09 | 57.31 |
| **10 μg/L** | 69.55 | 66.89 | 98.46 | 88.04 | 58.14 |
|  | 68.51 | 65.55 | 97.81 | 87.85 | 56.44 |
| **100 μg/L** | 73.33 | 70.24 | 97.93 | 87.73 | 56.5 |
|  | 73.55 | 70.39 | 97.85 | 87.54 | 56.33 |

**Table S4.** The log_2_(fold-change) values of 68 common DEGs in BPAF treatment groups.

|  | **0.1 μg/L** | **1 μg/L** | **10 μg/L** | **100 μg/L** |
| --- | --- | --- | --- | --- |
| ***LOC100006889*** | 5.97 | 5.86 | 6.55 | 9.42 |
| ***amfrb*** | 3.10 | 1.72 | 2.72 | 3.95 |
| ***LOC101886761*** | -5.37 | -4.83 | -5.29 | -5.37 |
| ***LOC110437733*** | 2.27 | 2.20 | 1.78 | 2.65 |
| ***her6*** | -2.03 | -2.04 | -7.51 | -2.02 |
| ***paqr3a*** | 2.01 | 1.37 | 1.77 | 1.71 |
| ***cand2*** | 8.94 | 9.50 | 8.58 | 9.35 |
| ***antxr2a*** | 1.59 | 1.70 | 1.01 | 1.41 |
| ***zgc:174888*** | 2.47 | 2.09 | 1.44 | 1.68 |
| ***itgb5*** | 2.83 | 4.37 | 4.83 | 3.23 |
| ***tmprss13b*** | 1.28 | 1.42 | 1.11 | 1.16 |
| ***si:ch211-89o9.4*** | 1.12 | 1.39 | 1.34 | 1.43 |
| ***agpat9l*** | 7.15 | 7.98 | 6.84 | 6.96 |
| ***egr4*** | -1.01 | -1.79 | -1.68 | -1.07 |
| ***gpr186*** | -1.07 | -1.43 | -1.53 | -1.37 |
| ***BGI_novel_G000059*** | 1.80 | 3.04 | -4.97 | 1.95 |
| ***BGI_novel_G000394*** | 2.55 | 1.48 | 3.46 | 3.46 |
| ***BGI_novel_G000540*** | 7.48 | 7.66 | 7.80 | 7.43 |
| ***BGI_novel_G000663*** | 1.11 | -7.00 | 2.12 | 1.44 |
| ***BGI_novel_G000758*** | 1.16 | 1.71 | 1.61 | 1.11 |
| ***BGI_novel_G000770*** | -7.91 | -7.93 | -7.84 | -1.58 |
| ***BGI_novel_G000862*** | -1.90 | -6.30 | -6.22 | -6.29 |
| ***BGI_novel_G000889*** | -1.80 | -1.88 | -1.48 | -1.70 |
| ***BGI_novel_G000921*** | 7.92 | 7.47 | 5.74 | 7.00 |
| ***BGI_novel_G001004*** | -9.68 | -1.34 | -2.41 | -2.35 |
| ***BGI_novel_G001055*** | 7.38 | 8.03 | 6.89 | 6.44 |
| ***BGI_novel_G001068*** | -9.25 | -9.26 | -9.18 | -9.26 |
| ***BGI_novel_G001387*** | -5.71 | -5.72 | -5.64 | -5.71 |
| ***BGI_novel_G001536*** | -1.20 | -9.68 | -1.15 | -9.67 |
| ***BGI_novel_G001581*** | -6.30 | -6.31 | -6.23 | 1.82 |
| ***BGI_novel_G001633*** | 5.22 | 5.20 | 7.54 | 5.06 |
| ***BGI_novel_G001752*** | 6.77 | 5.06 | 5.63 | 6.88 |
| ***BGI_novel_G001832*** | 5.89 | 4.84 | 5.93 | 5.96 |
| ***BGI_novel_G001878*** | 2.61 | 2.42 | 2.36 | 2.34 |
| ***BGI_novel_G001939*** | -1.75 | -1.98 | -1.20 | -1.78 |
| ***BGI_novel_G001992*** | -6.46 | -6.48 | -6.39 | -6.47 |
| ***BGI_novel_G002095*** | 7.20 | 4.78 | 5.09 | 6.17 |
| ***BGI_novel_G002138*** | 6.05 | 9.03 | 8.00 | 8.19 |
| ***BGI_novel_G002194*** | 1.18 | 1.53 | 2.67 | 1.58 |
| ***BGI_novel_G002201*** | -1.11 | -2.14 | -8.00 | -1.23 |
| ***BGI_novel_G002337*** | 8.15 | 6.18 | 7.38 | 7.20 |
| ***BGI_novel_G002405*** | 1.96 | -6.24 | 1.28 | -6.24 |
| ***BGI_novel_G002406*** | 1.28 | 1.10 | 1.04 | 1.42 |
| ***BGI_novel_G002415*** | 1.69 | 2.19 | 1.54 | -2.51 |
| ***BGI_novel_G002556*** | -7.51 | -7.52 | -7.44 | 1.01 |
| ***BGI_novel_G002560*** | 1.50 | -6.36 | 3.06 | -6.35 |
| ***BGI_novel_G002609*** | 4.36 | 6.64 | 5.57 | 6.61 |
| ***BGI_novel_G002691*** | 2.20 | 3.35 | 3.09 | 1.47 |
| ***BGI_novel_G002707*** | 1.75 | -4.29 | 1.80 | 2.35 |
| ***BGI_novel_G002730*** | 2.96 | 3.25 | 2.55 | 2.81 |
| ***BGI_novel_G002805*** | 2.89 | 3.23 | 3.28 | 2.80 |
| ***BGI_novel_G002812*** | -6.77 | -6.79 | -6.70 | -2.00 |
| ***BGI_novel_G002957*** | -2.34 | -1.27 | -2.40 | -1.27 |
| ***BGI_novel_G003004*** | -1.73 | -8.05 | -2.47 | -8.04 |
| ***BGI_novel_G003258*** | 2.54 | 1.63 | 1.55 | 1.53 |
| ***BGI_novel_G003302*** | -7.59 | -1.52 | -7.52 | -2.17 |
| ***BGI_novel_G003311*** | 7.93 | 5.54 | 6.51 | 5.73 |
| ***BGI_novel_G003326*** | -8.66 | -8.67 | -2.47 | -8.66 |
| ***BGI_novel_G003340*** | 1.75 | 2.03 | 2.03 | 2.62 |
| ***BGI_novel_G003349*** | 6.41 | 5.46 | 5.54 | 7.05 |
| ***BGI_novel_G003421*** | 8.68 | 10.55 | 9.87 | 9.19 |
| ***BGI_novel_G003499*** | 2.61 | 2.60 | 2.77 | 2.24 |
| ***BGI_novel_G003603*** | 8.19 | 9.05 | 7.61 | 8.15 |
| ***BGI_novel_G003672*** | 1.70 | -5.28 | -5.20 | 2.95 |
| ***BGI_novel_G003771*** | -7.36 | -2.19 | -7.29 | 2.03 |
| ***BGI_novel_G003781*** | 10.58 | 9.84 | 9.49 | 8.68 |
| ***BGI_novel_G003879*** | -5.93 | -5.94 | -5.75 | -5.93 |
| ***BGI_novel_G003955*** | -2.73 | 1.27 | 2.37 | 1.71 |

**Table S5.** The log_2_(fold-change) values of MAPK signaling pathway related genes in BPAF treatment groups.

| **Gene name** | **0.1 μg/L** | **1 μg/L** | **10 μg/L** | **100 μg/L** |
| --- | --- | --- | --- | --- |
| ***fgf3*** | -0.12 | -0.30 | -0.17 | -0.30 |
| ***fgf4*** | -0.05 | -0.55 | -0.52 | -0.85 |
| ***map2k1*** | -0.04 | -0.08 | -0.15 | -0.15 |
| ***myca*** | -0.20 | -0.41 | -0.42 | -0.48 |
| ***casp3b*** | 0.56 | 0.63 | 0.46 | 0.35 |
| ***vegfab*** | -0.02 | -0.12 | -0.24 | -0.17 |
| ***mycb*** | -0.35 | -0.66 | -1.17 | -1.22 |
| ***grb2b*** | -0.15 | -0.09 | -0.12 | -0.14 |
| ***mapk12a*** | 0.41 | 0.44 | 0.46 | 0.46 |
| ***arrβ2a*** | -0.08 | -0.25 | -0.21 | -0.24 |
| ***tek*** | -0.11 | -0.35 | -0.29 | -0.24 |
| ***tp53*** | 0.20 | 0.13 | 0.23 | 0.04 |
| ***csf1rb*** | 0.17 | 0.38 | 0.24 | 0.28 |
| ***fgf10a*** | 0.28 | 0.26 | 0.36 | 0.32 |
| ***flt4*** | 0.25 | 0.33 | 0.30 | 0.12 |
| ***LOC571431*** | -0.27 | -0.13 | -0.25 | -0.27 |
| ***dusp3a*** | -0.07 | -0.12 | -0.13 | -0.15 |
| ***rasa1a*** | -0.01 | -0.12 | -0.04 | -0.06 |
| ***si:ch73-63e15.2*** | 0.45 | 0.43 | 0.46 | 0.37 |
| ***cacng6b*** | 0.24 | 0.25 | 0.25 | 0.01 |
| ***si:dkey-246i14.3*** | 0.08 | -0.17 | -0.34 | -0.08 |
| ***map3k14a*** | 0.23 | 0.14 | 0.09 | 0.21 |
| ***tgfa*** | -0.08 | -0.07 | -0.15 | -0.12 |
| ***cacng7a*** | -0.05 | -0.21 | -0.32 | -0.30 |
| ***cacng6a*** | 0.10 | -0.18 | -0.11 | -0.18 |
| ***dusp10*** | 0.28 | 0.25 | 0.24 | 0.24 |
| ***flnca*** | 0.32 | 0.35 | 0.31 | 0.21 |
| ***tmem235*** | 0.85 | 0.39 | 0.80 | 0.26 |
| ***mapk8ip1b*** | -0.04 | -0.10 | -0.06 | -0.06 |
| ***si:dkey-11f12.2*** | -0.04 | -0.16 | -0.13 | -0.08 |
| ***mapk8ip1a*** | -0.03 | -0.13 | -0.11 | -0.07 |
| ***angpt2a*** | -0.72 | -0.25 | -0.79 | -0.72 |
| ***fgf9*** | -0.24 | -0.75 | -1.24 | -0.95 |
| ***LOC101884296*** | 1.23 | 1.78 | 1.26 | 1.17 |
| ***LOC101884923*** | -0.13 | -0.30 | -0.21 | -0.23 |
| ***LOC103909601*** | 0.10 | -0.25 | -0.54 | -0.14 |
| ***LOC103909948*** | 1.80 | 1.77 | 1.80 | 1.77 |
| ***LOC110438047*** | -0.13 | -0.38 | -0.25 | -0.25 |
| ***angpt1*** | 0.62 | 0.65 | 0.77 | 0.14 |
| ***dusp5*** | -0.09 | -0.13 | -0.27 | -0.47 |
| ***efna3b*** | -0.12 | -0.24 | -0.14 | -0.14 |
| ***pak2a*** | 0.22 | 0.15 | 0.09 | 0.04 |
| ***elk4*** | 0.14 | 0.31 | 0.17 | 0.30 |
| ***srfa*** | 0.27 | 0.26 | 0.22 | 0.18 |
| ***fgf8a*** | -0.04 | -0.13 | -0.23 | -0.25 |
| ***pla2g4aa*** | 0.41 | 0.52 | 0.29 | 0.52 |
| ***epha2a*** | 0.26 | 0.40 | 0.36 | 0.12 |
| ***igf2a*** | -0.08 | -0.15 | -0.70 | -0.51 |
| ***tgfbr2b*** | 0.28 | 0.27 | 0.25 | 0.13 |
| ***rap1b*** | -0.08 | -0.10 | -0.15 | -0.13 |
| ***pttg1ipa*** | 0.43 | 0.41 | 0.29 | 0.43 |
| ***nf1a*** | -0.01 | -0.10 | -0.13 | -0.04 |
| ***angptl1b*** | -0.05 | -0.22 | -0.02 | -0.36 |
| ***jun*** | -0.11 | -0.15 | -0.23 | -0.23 |
| ***rps6ka3a*** | 0.15 | 0.17 | 0.04 | 0.12 |
| ***fgf18a*** | -0.07 | -0.07 | -0.31 | -0.21 |
| ***fgf19*** | -0.21 | -0.86 | -0.35 | -1.35 |
| ***tgfb3*** | 0.00 | 0.06 | 0.03 | 0.14 |
| ***fgfrl1a*** | 0.23 | 0.29 | 0.29 | 0.15 |
| ***hsc70*** | 0.06 | 0.09 | 0.08 | 0.06 |
| ***prkacba*** | -0.23 | -0.03 | -0.40 | -0.42 |
| ***fosab*** | -0.35 | -1.97 | -2.25 | -1.93 |
| ***araf*** | -0.07 | -0.08 | -0.12 | -0.10 |
| ***LOC405768*** | 0.02 | 0.28 | 0.42 | 0.12 |
| ***cacna1sb*** | 0.18 | 0.30 | 0.25 | 0.20 |
| ***gadd45ba*** | -0.92 | -1.25 | -1.75 | -1.17 |
| ***dusp1*** | -0.16 | -0.58 | -1.00 | -0.90 |
| ***gadd45gb.1*** | -0.07 | -0.28 | -0.10 | -0.27 |
| ***plekhj1*** | -0.07 | -0.31 | -0.13 | -0.19 |
| ***rap1ab*** | -0.05 | -0.07 | -0.07 | -0.05 |
| ***mapk11*** | -0.19 | -0.34 | -0.39 | -0.23 |
| ***gadd45ab*** | -0.38 | -0.98 | -1.37 | -1.38 |
| ***arr3a*** | -0.18 | -0.24 | -0.26 | -0.31 |
| ***stmn4l*** | -0.04 | -0.09 | -0.33 | -0.20 |
| ***rac3a*** | -0.01 | -0.22 | -0.10 | -0.16 |
| ***kras*** | 0.08 | 0.12 | 0.05 | 0.14 |
| ***ppp3r1b*** | -0.02 | -0.06 | -0.07 | -0.07 |
| ***isoc2*** | 0.10 | -0.08 | -0.08 | -0.11 |
| ***fgfrl1b*** | 0.28 | 0.31 | 0.16 | 0.17 |
| ***gadd45bb*** | -0.71 | -0.84 | -1.38 | -1.01 |
| ***thap3*** | -0.02 | -1.15 | -0.89 | -0.63 |
| ***stmn1b*** | -0.08 | -0.15 | -0.17 | -0.14 |
| ***ccdc88c*** | 0.04 | -0.12 | -0.08 | -0.01 |
| ***map3k7*** | -0.01 | -0.08 | -0.17 | -0.05 |
| ***si:ch211-195b15.8*** | -0.19 | -0.22 | -0.34 | -0.24 |
| ***map4k3a*** | -0.21 | -0.09 | -0.09 | -0.28 |
| ***cpne4a*** | -0.03 | -0.11 | -0.13 | -0.13 |
| ***isoc1*** | 0.03 | -0.04 | -0.04 | -0.05 |
| ***mych*** | -0.45 | -0.88 | -1.66 | -1.70 |
| ***daxx*** | -0.14 | -0.19 | -0.07 | -0.19 |
| ***nfatc3a*** | 0.46 | 0.40 | 0.56 | 0.50 |
| ***flna*** | 0.19 | 0.22 | 0.25 | 0.16 |
| ***rac1b*** | 0.42 | 0.57 | 0.50 | 0.39 |
| ***map2k2a*** | 0.19 | 0.16 | 0.05 | 0.14 |
| ***nf1b*** | -0.01 | -0.10 | -0.03 | -0.04 |
| ***jund*** | -0.61 | -0.47 | -0.62 | -0.52 |
| ***insb*** | 2.14 | 0.85 | 2.43 | 1.00 |
| ***maptb*** | 0.00 | -0.10 | -0.09 | -0.11 |
| ***map3k7cl*** | 0.17 | 0.22 | 0.19 | 0.24 |
| ***il1rapl1a*** | -0.45 | -0.47 | -0.26 | -0.50 |
| ***fosaa*** | -0.61 | -0.68 | -1.09 | -1.23 |
| ***zgc:194887*** | 0.18 | 0.46 | 0.40 | 0.28 |
| ***fgf21*** | -0.26 | -0.29 | -0.90 | -0.43 |
| ***fam213ab*** | 0.06 | -0.40 | -0.25 | -0.07 |
| ***zgc:158417*** | -0.27 | -0.37 | -0.48 | -0.28 |
| ***ppp3ca*** | -0.07 | -0.14 | -0.12 | -0.10 |
| ***arr3b*** | -0.11 | -0.22 | -0.22 | -0.32 |
| ***LOC797032*** | -0.11 | -0.50 | -0.48 | -0.12 |
| ***cacng5a*** | 0.02 | 0.04 | 0.01 | 0.08 |
| ***stmn3*** | 0.07 | -0.10 | -0.03 | -0.02 |
| ***BGI_novel_G000190*** | 0.15 | -0.10 | -0.15 | -0.11 |
| ***BGI_novel_G000309*** | 0.36 | 0.24 | 0.02 | 0.13 |
| ***BGI_novel_G000375*** | 0.24 | 0.43 | 0.11 | 0.09 |
| ***BGI_novel_G000665*** | 1.28 | 2.65 | 1.95 | 1.39 |
| ***BGI_novel_G001219*** | 0.05 | -0.18 | -0.04 | -0.11 |
| ***BGI_novel_G001295*** | -0.22 | -0.58 | -0.11 | -0.41 |
| ***BGI_novel_G001339*** | 1.27 | 1.15 | 1.32 | -0.13 |
| ***BGI_novel_G001375*** | 0.17 | 0.13 | 0.06 | 0.14 |
| ***BGI_novel_G001377*** | -0.03 | 0.10 | 0.03 | 0.00 |
| ***BGI_novel_G001515*** | -0.42 | -1.51 | -1.46 | -0.67 |
| ***BGI_novel_G002253*** | 0.40 | 1.05 | 1.15 | -0.04 |
| ***BGI_novel_G002402*** | 0.20 | 0.17 | 0.06 | 0.02 |
| ***BGI_novel_G002856*** | -0.13 | -0.36 | -0.55 | -0.61 |
| ***BGI_novel_G003211*** | 0.40 | 0.77 | 0.33 | 0.78 |
| ***BGI_novel_G003275*** | -0.16 | -0.14 | -0.42 | -0.39 |
| ***BGI_novel_G003655*** | -0.23 | -0.06 | 0.80 | 0.08 |

**Table S6.** The mRNA level changes of selected genes in qRT-PCR results. All the data were presented by log_2_(fold-change).

| **Gene name** | **0.1 μg/L** | **1 μg/L** | **10 μg/L** | **100 μg/L** |
| --- | --- | --- | --- | --- |
| ***fgf3*** | -0.27 | -0.57 | -0.29 | -0.16 |
| ***fgf4*** | -0.26 | -0.80 | -0.59 | -0.39 |
| ***map2k1*** | -0.72 | -1.47 | -0.28 | -0.14 |
| ***myca*** | -0.10 | -0.62 | -0.30 | -0.07 |
| ***casp3b*** | 0.23 | 0.19 | 0.31 | 0.95 |
| ***grb2b*** | -3.08 | -1.40 | -0.08 | -0.23 |
| ***mapk12a*** | 1.00 | 1.00 | 1.41 | 1.46 |
| ***arrβ2a*** | -0.10 | -0.27 | -0.17 | -0.02 |
| ***tek*** | -0.34 | -0.01 | -0.16 | -0.12 |
| ***tp53*** | 1.08 | 0.74 | 0.82 | 1.32 |
| ***csf1rb*** | 0.24 | 0.22 | 0.29 | 0.33 |
| ***fgf10a*** | 1.30 | 0.68 | 2.24 | 2.17 |
| ***flt4*** | 0.35 | 0.02 | 0.37 | 0.52 |
| ***LOC571431*** | -2.26 | -2.75 | -2.23 | -1.55 |
| ***vegfab*** | -0.10 | -0.38 | -0.17 | -0.10 |
| ***mycb*** | -0.14 | -0.76 | -0.92 | -0.82 |


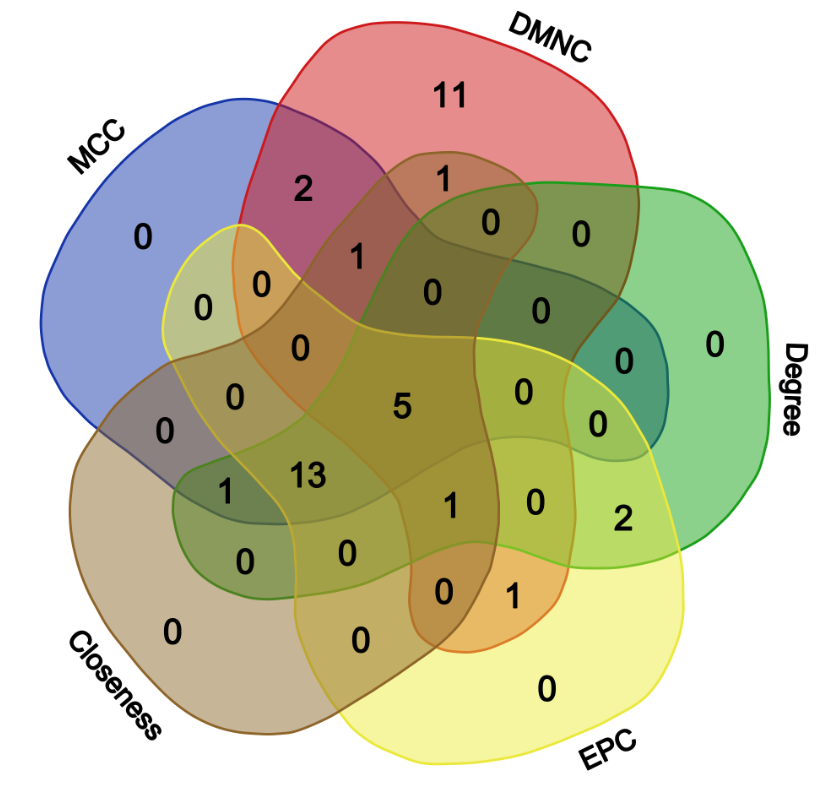


**Fig. S1.** Venn diagram of the top 22 hub genes in five algorithms including MCC, DMNC, EPC, Closeness, and Degree.
